# Prosthetic Valve Endocarditis After Y-Incision Aortic Annular Enlargement: A Simple Solution

**DOI:** 10.1016/j.atssr.2024.06.004

**Published:** 2024-06-24

**Authors:** Kanhua Yin, Katelyn Monaghan, Bo Yang

**Affiliations:** 1 Department of Cardiac Surgery, University of Michigan, Ann Arbor, Michigan

## Abstract

The Y-incision aortic annular enlargement (AAE) has been established as a safe and effective technique for upsizing the aortic annulus by 3 to 4 valve sizes. However, concerns have been raised regarding its technical complexity during reoperations, particularly given the extensive enlargement of the aortic annulus and root. We present a case of reoperative aortic valve replacement after previous Y-incision AAE for prosthetic valve endocarditis and aortic root abscess. Our case highlights the simplicity and effectiveness of using a rectangular patch for root reconstruction and implanting the “roof” technique for aortotomy closure in reoperations after Y-incision AAE.

The Y-incision aortic annular enlargement (AAE) technique, initially developed in 2020, has undergone continuous refinement.^1^, ^2^, ^3^, ^4^ Promising early outcomes from 119 consecutive cases reveal an average increase in the annular size of 3 to 4 valve sizes, with few postoperative complications, including 1 operative mortality, 1 stroke, and 2 pacemaker implantations (1 pacemaker was implanted for a patient with aortic valve endocarditis complicated by a Gerbode fistula).^3^ Y-incision AAE has been widely adopted globally and is used in treating diverse aortic root disorders, including congenital aortic stenosis in pediatric patients.^5^ Recognizing the likelihood of subsequent cardiac operations in a patient’s journey, it becomes crucial to establish a simple and effective approach for reoperation, especially in challenging scenarios such as when normal anatomic structures have been destroyed by endocarditis, as described in a recently published case report.^6^ In this context, we present a simple technique of using a rectangular patch for root reconstruction and the “roof” technique for aortotomy closure to address prosthetic valve endocarditis and root abscess after Y-incision AAE.

## Technique

A 68-year-old woman underwent aortic valve replacement and Y-incision AAE with enlargement of the aortic annulus from 23 mm to a size 29 Magna Ease valve (Edwards Lifesciences) for severe aortic stenosis. At 3-month follow-up, computed tomographic angiography revealed no pseudoaneurysm, and the echocardiogram showed a mean gradient of 7 mm Hg and a normal appearance of the prosthetic valve and aortic root. However, 6 months after the index operation, she presented with prosthetic valve endocarditis, which likely resulted from chronic cellulitis and colonization of methicillin-resistant *Staphylococcus aureus*. Transesophageal echocardiography revealed a 20 × 14 mm paravalvular abscess, necessitating reoperation.

**Figure 1:**
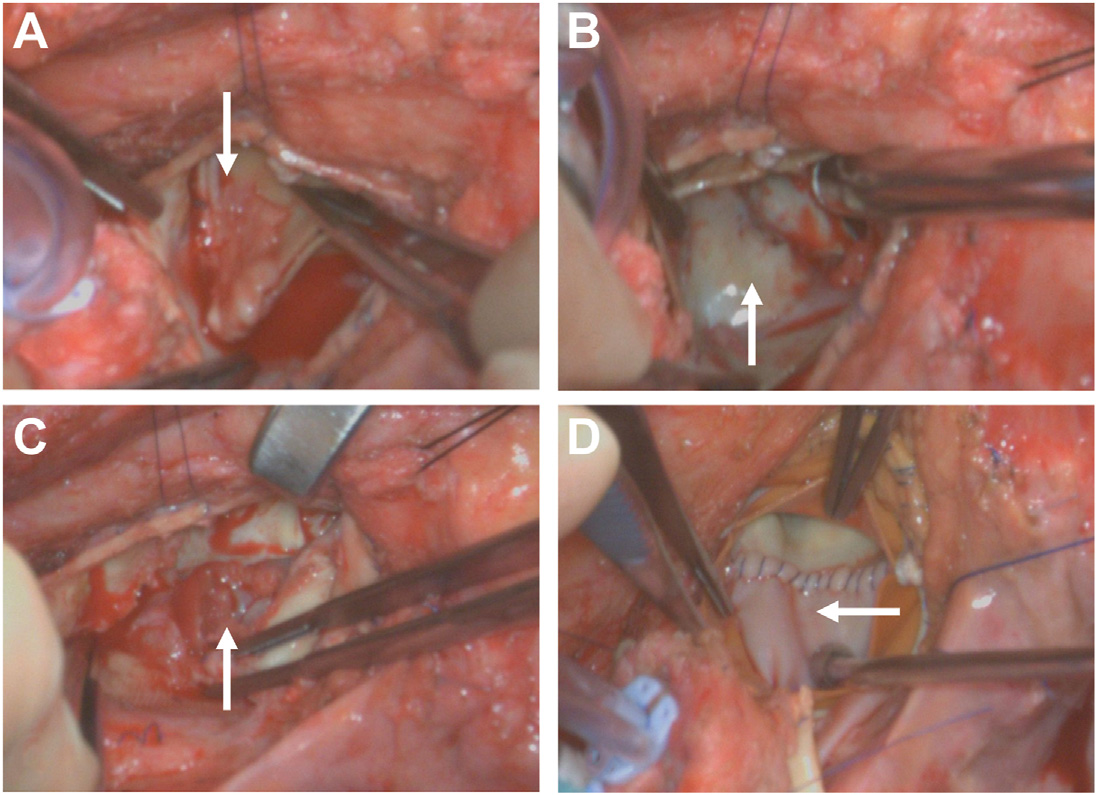
Intraoperative findings. (A) Vegetations (arrow) observed on the left coronary cusp. (B) The rectangle Hemashield patch (Getinge) placed during the index operation was fully epithelialized beneath the prosthetic valve (arrow), thus forming the new aortomitral curtain. (C) Abscess (arrow) identified behind the patch, located above the dome of the left atrium. (D) A new bovine pericardial patch was anastomosed to the aortomitral curtain (arrow) during the reoperation after Y-incision aortic annular enlargement.

The ascending aorta was transected at the previous aortotomy suture line. Vegetations were observed on the left coronary cusp (Figure 1A). Complete epithelialization was noted over the Hemashield patch (Getinge)—now serving as the new aortomitral curtain—below the previous prosthetic aortic valve (Figure 1B). An abscess was identified above the dome of the left atrium behind the Hemashield patch underneath the prosthetic valve (Figure 1C). Dehiscence was observed in the patch from the aortic annulus at the nadir of the noncoronary sinus. The patch was carefully dissected off from the left atrium to the level of anterior mitral annulus without much difficulty. The abscess cavity was thoroughly debrided. A new rectangular bovine pericardial patch, trimmed to the appropriate size, was anastomosed to the anterior mitral annulus from the left fibrous trigone, the right fibrous trigone, and then to the remnant aortic annulus of the noncoronary sinus and the left coronary sinus (Figure 1D). A size 29 Magna Ease valve was soaked in rifampin and implanted as the index Y-incision AAE, as previously described.^2^^,^^3^ The aortotomy was closed using the roof technique, with an additional 2-cm posterior longitudinal aortotomy at the posterior ascending aorta.^2^^,^^3^ The Video provides a visual guide to the key technical details of reoperation after Y-incision AAE.

**Figure 2:**
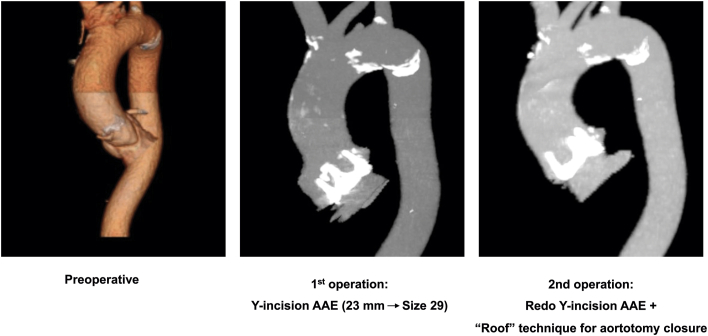
(Left) Preoperative, (middle) after post–index Y-incision aortic annular enlargement (AAE), and (right) postreoperation after Y-incision aortic annular enlargement computed tomographic angiography images. Postreoperation computed tomographic angiography demonstrates a well-seated size 29 valve with the “roof” technique used to close the aortotomy.

The patient did well postoperatively and was discharged without complications. Computed tomography angiography at 5 months displayed a well-seated bioprosthetic aortic valve and an excellent contour of the aortic root and ascending aorta (Figure 2). At 2-year follow-up, the echocardiogram indicated a mean gradient of 11 mm Hg and a normal prosthetic valve with no endocarditis recurrence. Written informed consent was obtained from this patient.

## Comment

Given the extensive root enlargement in Y-incision AAE, managing prosthetic valve endocarditis complicated by root abscesses can be challenging. In our patient, a rectangular patch was used to reconstruct the aortic root after thorough debridement of the abscess cavity, similar to the index Y-incision AAE. Regarding patch selection, we elected to use a bovine pericardial patch, considering the patient’s prosthetic valve endocarditis. The patient reported here remained infection-free during 2 years of follow-up. In another native valve endocarditis case, we used a rifampin-soaked Hemashield patch, which also achieved excellent outcomes. For patients with extensive root damage secondary to infective endocarditis, we generally recommend total root replacement with a homemade composite graft or Freestyle aortic root (Medtronic).^7^ Removing the previous patch in patients without prosthetic valve endocarditis is unnecessary, and a large new valve can be easily placed in the enlarged aortic annulus. Conversely, in this case, we observed complete epithelialization and no thrombus formation on the previous Hemashield patch under the prosthetic valve, which served as the neoaortomitral curtain. These findings support the safety of using the Hemashield patch for the index Y-incision AAE. Finally, the roof technique emerged as a valuable strategy for enlarging the proximal ascending aorta, thereby preparing the patient for future valve-in-valve transcatheter valve replacement.

In conclusion, prosthetic valve endocarditis after a Y-incision AAE with a root abscess could be managed by thoroughly debriding the root abscess and using a new rectangular patch for root reconstruction and aortic valve replacement, just as in the initial Y-incision AAE.
